# A Theoretical Approach to Electronic Prescription System: Lesson Learned from Literature Review

**DOI:** 10.5812/ircmj.8436

**Published:** 2013-10-05

**Authors:** Mahnaz Samadbeik, Maryam Ahmadi, Seyed Masoud Hosseini Asanjan

**Affiliations:** 1 Department of Health Information Management, Institute of Health Management and Information Sciences, Iran University of Medical Sciences, Tehran, IR Iran; 2 Department of Health Information Technology, Lorestan University of Medical Sciences, Khoramabad, IR Iran; 3 Department of Medical Informatics. Institute of Advanced Medical Technologies, Tehran University of Medical Sciences, Tehran, IR Iran

**Keywords:** Electronic Prescribing, Utilization, Standards

## Abstract

**Context:**

The tendency to use advanced technology in healthcare and the governmental policies have put forward electronic prescription. Electronic prescription is considered as the main solution to overcome the major drawbacks of the paper-based medication prescription, such as transcription errors. This study aims to provide practical information concerning electronic prescription system to a variety of stakeholders.

**Evidence Acquisition:**

In this review study, PubMed, ISI Web of Science, Scopus, EMBASE databases, Iranian National Library Of Medicine (INLM) portal, Google Scholar, Google and Yahoo were searched for relevant English publications concerning the problems of paper-based prescription, and concept, features, levels, benefits, stakeholders and standards of electronic prescription system.

**Results:**

There are many problems with the paper prescription system which, according to studies have jeopardized patients’ safety and negatively affected the outcomes of medication therapy. All of these problems are remedied through the implementation of e-prescriptions.

**Conclusions:**

The sophistication of electronic prescription and integration with EHR will become a reality, if all its stakeholders collaborate in developing fast and secure electronic prescription systems. It is plausible that the required infrastructure should be provided for implementation of the national integrated electronic prescription systems in countries without the system. Given the barriers to the implementation and use, policymakers should consider multiple strategies and offer incentives to encourage e-prescription initiatives. This will result in widespread adoption of the system.

## 1. Context

Medication prescription is one of the most common and powerful treatment methods used by physicians. For years, hand-written prescription has been a preferred communication method for physicians in decision making concerning medication therapy, and pharmacists in in distributing medications. On the other hand, it has been a valuable instruction on how to use medications for patients. Besides, it is considered as an important activity in the health care process (1-4). National health care systems face numerous forces such as demographic changes followed by increasing need for health care services. Hence, people tend to use more medications which have been resulted in rising the number of prescriptions and diversity in medication types. Therefore, society became much more reliant on medication prescribing, distributing, administrating and processing systems (5-10).

Medication process is an erroneous procedure. Among different types of errors associated with medication process, prescription errors are the most preventable cause of medication errors. Traditional hand written prescription is too slow due to using pen and paper. The paper-based prescription process is inefficient, expensive and resource-intensive. This approach has several other limitations such as high rate of human errors in manipulating data, and documentation errors which are inevitable (11-16). However, it is so hard to overcome the limitations of paper prescription that arise due to the growing number of pharmaceutics and complexity of medical care. Most of the limitations of paper prescription could be eliminated or minimized by electronic prescription or e-prescription (e-Rx)(14, 17-19). Hence, electronic prescription has emerged as a viable and definitive solution to counter shortcomings of the current paper-based prescription system, in terms of fraud, inefficiency and administrative workload. The prevalence of electronic prescription is facilitated by the impressive progress in information and communication technology (ICT), the widespread use of computer in health care, and the continuing decrease in the price of computer technology (13, 20-28). This approach improves the quality and reduces the growing costs of health care services (27, 29, 30). In general, medication prescription is an important and vital process in modern health care, and implementation of electronic prescription systems provides numerous functionalities for more effective and efficient prescription (5, 11-16, 31). As we did not identify literature objectively reporting the current knowledge on electronic prescription systems, this narrative literature review is intended to link together many studies on different themes concerning topic areas for purposes of interconnection, which may be helpful to a variety of potential stakeholders, including health care providers, pharmacist, health information technologist and health policy makers.

## 2. Evidence Acquisition

**Figure 1.**
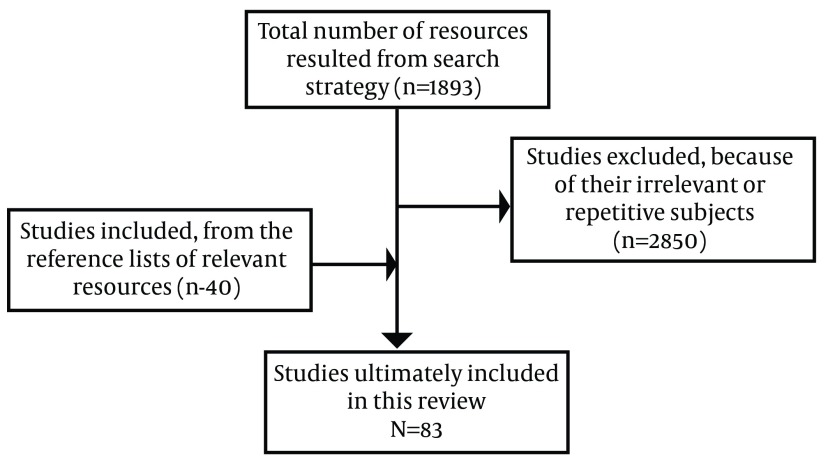
The Steps of Resources Selection

We searched following search engines and databases: Yahoo, Google, Google Scholar, PubMed, ISI web of science, Scopus, EMBASE, Iranian National Library Of Medicine (INLM) by a group of Mesh terms and keywords pertaining to electronic prescription. The original list of search terms used in this review is presented in Table 1. The searches were done by using Boolean operators OR, AND between main phrase and the mentioned keywords were extracted from specific themes of the topic under study. Beside articles, extracted guides, blueprints, manuals and reports were also included. Moreover, reference lists of all relevant resources were interrogated as a part of the search strategy. The search strategy was limited to English language resources with no time limitation. All articles and manuals describing concept, characteristics, levels, advantages, stakeholders and standards of electronic prescription system were included. Key terms of Paper-based prescription were not included in search strategy and problems related to the paper-based prescription systems were extracted from the final selected resources. The resources were selected through consensus of research team members and all included papers were reviewed by the first author of the article. The search for resources related to E-Prescription terms took place between Oct 1, 2011 and Feb 12, 2012 and resulted in 1893 resources. The resulting resources were evaluated, based on their relevance to this study. This step resulted in the exclusion of 1850 resources which were out of scope and inclusion of 40 studies from reference lists of retrieved resources. Finally, 83 studies identified as relevant. Figure 1 summarizes the steps taken to select relevant resources. 

**Table 1.**
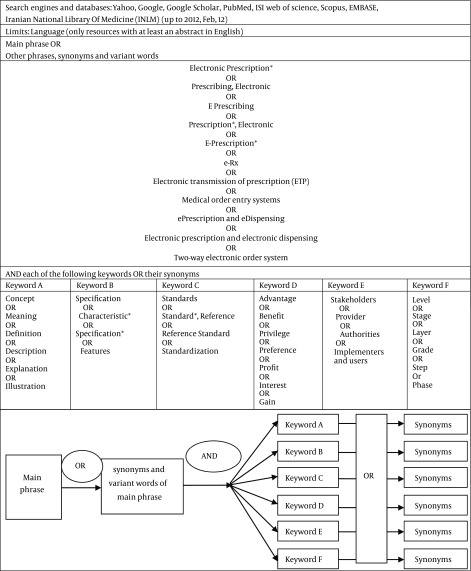
Search Strategy Table

## 3. Results

### 3.1. Problems Related to the Paper-Based Prescription Systems

There are many problems with the paper prescription system which, according to studies have jeopardized patients’ safety and negatively affected the outcomes of medication therapy. All of these problems are remedied through the implementation of e-prescriptions. Examples of prescription problems are mentioned below:

• Errors in drug name, dose, formulation, frequency of dosing, dosing regimen, strength and rout

• Illegibly written prescriptions

• Ambiguous order and incorrect interpretation of the prescription

• Unclear telephone or verbal orders

• Prescriptions issued to the wrong patient

• Missing prescriber or patient data

• Omission of medication, high rate of prescription fraud imposing costs on pharmacies

• Rewriting prescriptions in physicians’ offices and pharmacies

• Re-entering all the prescription details into pharmacy and paying system by hand

• Giving potential rise to re-keying errors

• Incompleteness of information on patient medication histories

• Repetitive medication treatment

• Miscommunication due to illegible handwriting in ordering, distributing and administering of medications

• Unclear abbreviations and dose designations

• Poor tools for managing adverse drug interactions

• High rate of adverse drug reactions

• Complexity of medication selection due to wide variety of pharmaceutical products

• High cost for handling prescriptions

• Risk of losing, damaging and hefting a paper prescription

• Having difficulty obtaining accurate patient-specific formulary information (7, 11, 14, 17, 24, 32, 33).

**Figure 2.**
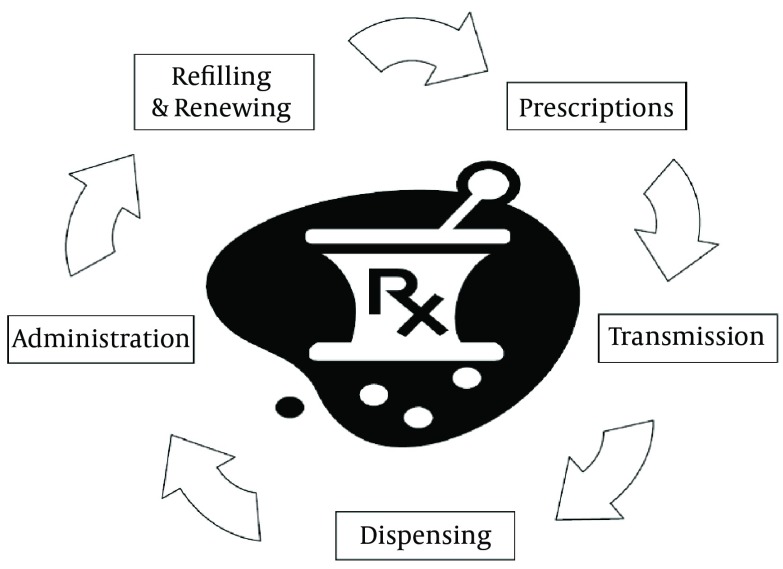
Paper Prescription Cycle Sands200834(34)343412Sands, D. Z.E-prescribing: what is It? why should i do It? what's in the future?2008Washington DCAmerican College of Physicians Internal Medicine http://www.acponline.org/running_practice/technology/eprescribing/mtp097.pdf (34)

In paper prescription cycle demonstrated in figure 2, the prescription is written by a physician. Then, the patient receives a written prescription and transmits it to a pharmacy. The prescribed medications are distributed at the pharmacy and patient picks up the prescription from a pharmacist. Finally, the medication is administered to the patient. After termination of the treatment period, patient should refer to the physician to pick up a paper prescription for Rx refilling or renewal if it is needed (26, 29, 34). Therefore, electronic prescription is suggested to improve proper medication adherence, access to medication history and prescription benefits at the point of care, provide electronic communication between prescribers, pharmacies and health programs/health insurances (5, 31, 33, 35).

### 3.2. Electronic Prescription

#### 3.2.1. The Concept of Electronic Prescription

Electronic prescription is a reality far beyond the simply using computers to write and save prescriptions (36). In fact, electronic prescription (e-prescription) is a broad term that means using the computer devices to enter, modify, review and generate or transmit medication prescriptions that prepare two-way transmissions between the point of care and the dispenser. This form of technology would safely transmit prescription or prescription-related information between stakeholders (prescribers, dispensers, pharmacies, health plans, and health insurers) either directly or through an intermediary (including an electronic prescription network) using electronic media. E-prescription transfers prescriptions from prescribers to pharmacies, refills and renewals requests from pharmacies to providers, prescription benefit and formulary information and fills status notification for prescribers. Therefore, the utilization of electronic systems in prescription can facilitate the communication of a prescription, aid the choice, and supply the medication by decision support and finally provides a robust audit trail for the entire medication process (37-42)

**Figure 3.**
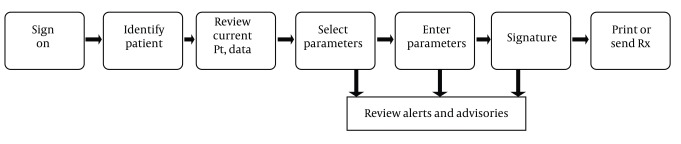
Process for Creating and Managing a Prescription Electronically (38)

**Figure 4.**
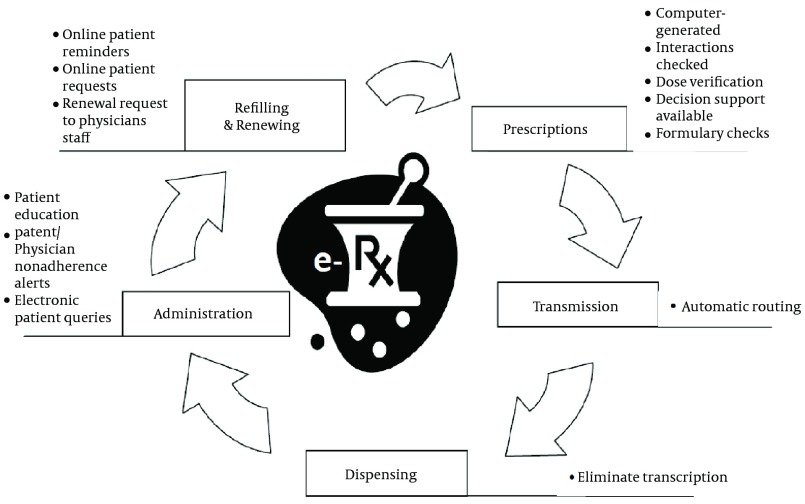
E-Prescription Cycle Sands200834(34)343412Sands, D. Z.E-prescribing: what is It? why should i do It? what's in the future?2008Washington DCAmerican College of Physicians Internal Medicine http://www.acponline.org/running_practice/technology/eprescribing/mtp097.pdf (34)

There are several main steps in creating and managing prescriptions electronically, as depicted in Figure 3. Firstly, a user of the system signs in by some sort of authentication to prove his or her identification. In the next step, a clinician identifies a patient within the electronic prescription system and the electronic prescription process begins. These data should be readily available to the clinician prior to entering new prescriptions. Different devices in multiple environments are used in three activities of the electronic prescription process, such as selecting a medication, entering parameters and signing the prescription. Also, clinical decision support is utilized through reviewing alerts and reminders in these activities. Then, the verified prescription was directly or indirectly transferred to pharmacy for dispensing. Prescription Refill and renewal requests are also automated in e-prescription cycle, illustrated in Figure 4. Moreover, prescription claims are transmitted electronically from pharmacy to payers (7, 36, 38 - 42).

#### 3.2.2. Features of Electronic Prescription System

A desirable electronic prescription system should meet the following features and functionalities:

• Producing prescription electronically

• Generating a complete active medication list

• Selecting medications, printing prescriptions, electronically transmitting prescriptions, delivering clinical knowledge to the prescriber, and controlling all patients’ safety based on the patients’ demographics and medical history to support decision-making and knowledge at point of care, (safety controls include automatic alerts and reminders representing data about ordering correctness, inappropriate dose or route of administration of a drug-drug interactions, drug allergies, drug or diagnostic test contraindications, age-specific warnings, or cautions)

• Providing information on the availability of lower cost medications and medically appropriate treatment alternatives

• Providing information on formulary, co-pay (or coinsurance) and patients’ eligibility received electronically from the patient’s medication plan

• Facilitating electronically transfer of prescription to the pharmacy by sending messages and using the interoperable standards

• Speeding up the process of renewing medications with ability to send prescription renewal requests electronically and automate the renewal authorization process

• Supporting patients during administration and actively promoting the appropriate medication usage

• Linking to other elements of patients’ health records

• Making improvements of work processes

• Provision of instructions and educational materials for patients and clinicians 

• Providing powerful evaluation instrument for the entire medicines use process (42-45)

#### 3.2.3. Levels of Electronic Prescription

**Figure 5.**
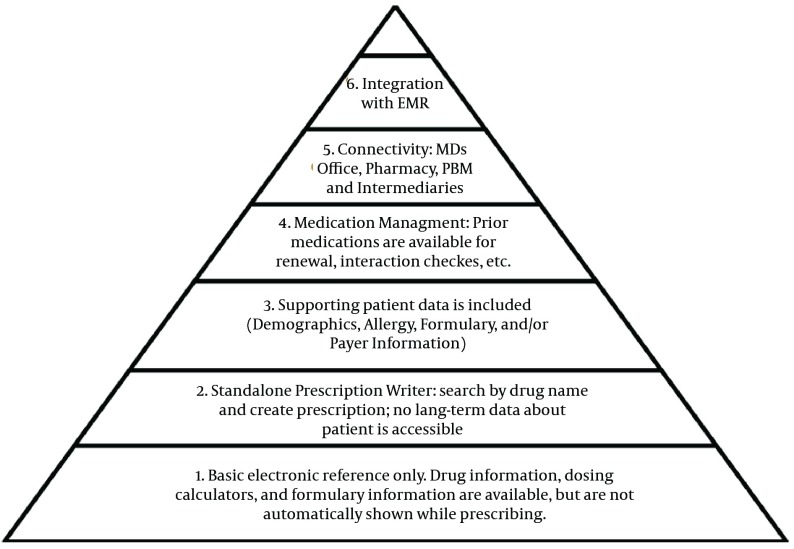
Graduated Levels of Electronic Prescription (38)

E Health Initiative (EHI) has outlined six different graduated levels of e-prescription from basic reference systems to advanced systems demonstrated in a pyramid (figure 5). Each level covers more functionalities than previous one. The levels of the pyramid are:

1. Electronic prescription references only, no prescription capability

2. Stand-alone prescription writer, with no medication history or supporting data

3. Addition of basic supporting data, such as allergies, demographics, and formulary information, which can be used by the system to generate alerts 4. Medication management – long-term tracking and monitoring of each patient’s active medications

5. Connecting the practices, pharmacies, payers, pharmacy benefit managers (PBM’s), intermediaries, and patients

6. Integration with a more complete electronic health record.

Advantages related with this system are seen in all above mentioned levels. Nevertheless, systems at the higher levels of sophistication (which have cost of implementing) provide much better opportunities for improving quality, reducing errors, and improving workflow efficiency. However, these advantages are accessible in lower levels e-prescription systems as well. Higher levels and benefits are available through more associated information about patient, data resources in the prescription chain and better relationship among stakeholders involved in the medication management process. Electronic Prescription can be initiated from mid-levels based on existing resources and needs. Then, the level can be enhanced by adding more capabilities and features. The final goal is to reach the highest levels with the most advantages (20, 37, 40, 41, 45).

Another five-stage model is also created by e-Health Observatory (46) to subdivide e-prescription functionality, which is based on the EMR Adoption Model presented by HIMSS Analytics (47), and includes zero stage of paper prescription environment to the fifth stage of complete implementation of electronic prescription and link with the electronic medical record (EMR). In this model, some new processes do enter the workflow with increasingly sophisticated e-prescription systems. A summary of each stage features is indicated in Table 2. 

**Table 2.** Facilities and Features of Each Stage (46)

Stage	Description
**Stage 0**	Paper-based prescription environment
	Pharmaceutical coverage requests are completed by hand.
**Stage 1**	Medication information is captured in free text and stored digitally.
	Pharmaceutical coverage requests are done on-computer and then printed.
**Stage 2**	Medication lists and renewals are generated from the EMR.
	Prescriptions are generated by the EMR, but still printed or faxed.
**Stage 3**	The EMR’s e-Prescription system maintains a medication list for all patients.
	System supports some capability of decision support.
	Prescriptions are generated by the EMR, but still printed or faxed.
	Renewal request is supported within the EMR, but still received by phone or fax.
	Pharmaceutical coverage requests are completed within the EMR, but still sent by fax.
**Stage 4**	Enhanced patient-specific alerts include in this stage.
	The system allows clinician to access a list of favorite medications, and offers assistance re-dosing based on patient-specific criteria.
	Insurer coverage criteria are embedded within the EMR.
	Forms can be generated and sent within the EMR and approval/rejection is automatically linked back to the patient.
**Stage 5**	Full circle e-prescription without paper in between, including the writing of prescriptions, but also the renewal of requests from pharmacists and syncing of medication lists with regional repositories to better support clinical decision support.
	The pharmaceutical coverage process is linked within the EMR as part of a region-wide e-Prescribe system where application, approval, and subsequent prescription generation are all part of the system.

#### 3.2.4. Advantages of Electronic Prescription

Implementing electronic prescription system can overcome many problems related to paper prescription process; and offer the following advantages:

• Cost savings and reduced expenses of health care for patients, health care providers, health programs, and health care insurance organizations throughout the prescription processing system (48-52).

• Reducing prescription, medication and transcription errors, adverse drug reaction (ADR), prescription fraud and litigation (48, 50, 53-61).

• Improving medication therapy outcomes, patient safety, practice, health care efficiency and quality, and public health (52, 59, 62)

• Providing a safe instrument for electronic access to updated formulary information and patient medication history at the point of care

• Supporting clinical decision-making for medication therapy in the context of drug-drug interactions, drug-allergy reactions, diagnoses, duplicative treatment and dose calculation based on age, weight, health history, and etc.

• The possibility of receiving the prescription electronically from physicians and refilling or renewing the medication prescriptions by pharmacy

• Increasing patient convenience and medication compliance

• Reducing redundant paperwork

• Increasing legibility, preciseness, correctness, and completeness of prescriptions

• Easing the sharing knowledge for better patient care and reducing improper verbal relationships

• Providing a safe, two-way, and electronic communication between all stakeholders and Supporting coordinated inter and intra-team work

• Establishment of a safe national infrastructure for data communication

• Reforming clinical work flows associated with medication management

• Enhancing patient satisfaction (2, 5, 27, 33, 37, 40, 41, 50, 63-67)

#### 3.2.5. Stakeholders in Electronic Prescription

Electronic prescription system has the following various stakeholders who play significant role in complex process of creating and managing prescriptions:

• Patients: consumer, patient groups, family caregivers, patient representatives, and advocacy groups

• Health care providers: prescribers (clinical specialists or general practitioners), practice organizations, hospitals, clinics, long-term care facilities, hospices, managed care organizations, dispensers (pharmacists and pharmacy staffs), academic and research health institutions and medical groups

• Health care payers: employers, health plans, third-party payers, insurances providers, patient supporting programs

• Pharmacy networks: community, retail and mail order pharmacies 

• Healthcare information technology producers/suppliers for outpatient and acute care settings

• Policy makers: federal and state representatives, congresses, legislators, medical boards, boards of pharmacy, community-based health information exchange collaborative, standards development organizations

• Other interested Parties: medical societies, nursing associations, academic and research institutes, agencies tasked with combating medication smuggling and use, pharmaceutical production factories, other groups involved in exchanging or receiving such electronic health information as medication history ( 29 , 43 , 68 - 70 ). As it is indicated in Table 3, most values of electronic prescription system will be for health care providers. Meanwhile, the patient safety and healthcare affordability are valuable for all main groups of stakeholders. 

**Table 3.** Values of Electronic Prescription for Main Stakeholders (70)

	Patients	Providers	Pharmacies	Payers / PBMs / Public Sector
**Enhanced patient-specific and drug reference information at point of care**	√	√		
**Patient Safety**	√	√	√	√
**Efficiency/Productivity**		√	√	
**Healthcare Affordability**	√	√	√	√
**Convenience**	√	√		

#### 3.2.6. Standards of Electronic Prescription Systems

In order to share critical information contained in medication prescriptions across disparate health care settings, systems from multiple vendors must be able to readily inter-operate and exchange prescription data. The inability to share information across the multiple systems with a standard format and expression has been a major hindrance to improve medication prescription process, which results in increased costs, redundancies and inefficiencies. Therefore, the need for the standards of electronic prescription has become increasingly evident. The six principal e-prescription standards include:

1. Medication history standard, intended to provide a uniform means for prescribers, dispensers, and payers to communicate around the list of medications that have been dispensed to a patient. At the time of prescription , the access to the patient's past prescription provided by pharmacies and prescription insurances can help to reduce and prevent adverse drug events (ADEs) due to the interaction of new medication with existing active medications and allergies of the individual and also improves the quality of care and patients’ safety.

2. Formulary and benefits standard, intended to provide prescribers with information about a patient’s medication coverage at the point of care, and informs prescribers of lower cost medications for patient. Adoption of this standard added value for estimating the patient’s medication coverage and lead to patient-specific, real-time benefit information and better formulary compliance. Information may include whether medications are considered to be "on the formulary," or offthe formulary. Rules for prior authorization and step therapy, and the cost of medication for the patient, and minimizing the need for phone calls between pharmacy and physician’s office are the issues considered in benefit standards 

3. Prescription Fill Status Notification (RXFILL) standard, serving the purpose of notifying the prescriber about the status of a newed or refilled prescription. This transaction is originated by the pharmacy and can be used to notify in three cases, when Rx is filled, not filled, or partially filled. So that compliance or non compliance of patient could be estimated, and helps health care providers to control patients with chronic diseases such as diabetes and hypertension via this function.

4. Prior authorization standard, which refers to any process that requires obtaining approval or certification from a health insurers, that the patient meets criteria for a medication to be covered. This precertification requires header information, the requester, subscriber, utilization management, and other relevant information for pre-authorization of requests.

5. Structured and Codified SIG standard, describes patient instructions for taking medications that are currently expressed as free text at the end of a prescription (called the signature, commonly abbreviated SIG), including indication, dose, dose calculation, dose restriction, route, frequency, interval, site, administration time and duration, and stop order instructions. A standard structure and code set for expressing SIGs would enhance patient’s safety. Currently, there is no standardized format or expression for SIGs, leaving room for misinterpretation and error.

6. Clinical drug terminology standard, provides standard names for clinical medications (active ingredient, strength and dosage form) and dose forms as administered to a patient. RxNorm standard, a clinical drug nomenclature that also provides links from clinical drugs to their active ingredients, drug components, and most related brand names, is expected to face the problem of the existence of currently multiple databases of drug names, forms, and dosages.

7. Standard provider identifier, proposing a unique numeric identifier assigned to healthcare providers and organizations. The use of national identification number in all electronic prescription transactions can accelerate prescription workflow and eliminate call backs from the dispenser to identify prescribers (41, 43, 45, 71-74).

## 4. Conclusions

Strengthening the health systems is complex and demanding. Nowadays e-Health has emerged as a fast-growing technology and effective tool to meet day-to-day healthcare needs. In this regard, the pace of using information technology in prescription and dispensing medications to improve the quality, safety, and health care efficiency has been rapidly accelerated in recent years. Through the use of computerized systems, electronic prescription systems provide the potential to dramatically improve the outcome of prescription processes, reduce adverse drug events, and save health care costs. Strengthening and supporting electronic prescription system requires the collaboration between all system stakeholders and the establishment of the underlying healthcare information infrastructure, which leads to better health care for every person of the society. In this way, the integrated electronic prescription system would become a reality. Regarding final approval of many technical standards about the integrity of data transmission as well as verification of security, privacy, and interoperability of many electronic prescription products, it is vital and essential to establish information exchanges with national e-prescription networks. It can improve health care quality, reduce cost and save people’s lives. Given the barriers and problems of implementation and use of e-prescription systems, policymakers should provide the multiple strategies to encourage widespread adoption and long range planning necessary for a successful change management process. Therefore, it is required to provide the necessary arrangements for implementing the national electronic prescription system and having access to electronic drug references in countries without such systems. Some fundamental preparations for this system are required including providing appropriate infrastructure for handling electronic prescription, evaluating workplaces, defining stakeholders' needs, educating and supporting main actors, funding required expenses and discarding the traditional methods of medication prescription and dispensing. In addition, in developed countries, proper planning should be done by governors and legislators to overcome the major problems of current electronic prescription systems such as concern about Return of Investment (ROI), the low rates of adoption of health among stakeholders due to possible changes in current workflows, dual workflows, the need to maintain paper- and fax-based prescription for controlled substances, threat to the autonomy of the physician's clinical decision making capacity, old and inflexible technology infrastructure, data availability and complexity of the selection process of hardware and software for implementing e-prescription systems (40, 67, 75). Moreover, additional payment incentives or other financial support would be required to eliminate substantial barriers to e-prescription adoption and use by physicians including maintaining complete patient medication lists, using clinical decision support, obtaining formulary data, and electronically transmitting prescriptions to pharmacies (36). In short, this health care information system can manage the challenges of increasing medication costs and meet the demands of patients .

### 4.1. Recommendations and Suggestions

The related studies performed in Iran (76-80) have highlighted a high frequency of medication error, quantitative and qualitative problems and imperfections in the current prescription system. Moreover, these studies have emphasized on the impact of computerized provider order entry (CPOE) on reducing medication error and improving patient safety, and also the patients' willingness to refill their prescriptions electronically and obtain more information about the administration of medication via information technology.

Nowadays, Some great opportunities have been provided on medication prescription in Iran, including the possibility of entering medication request in hospital information systems, designing applications for management of health care settings, using smart card for specific groups of patient, initiating the online verification of paper prescriptions and Online acceptance of medication prescriptions from pharmacies by social security organization (81-83). Therefore, it is necessary to give a high priority to designing and implementing a national, unified and safe electronic prescription system by Iranian health care system.
